# Supplementary material for: Clinical status and cytokine profiles in patients with asthma or chronic obstructive pulmonary disease vaccinated against influenza
Source: PLoS One. 2025 Feb 12;20(2):e0313539. doi: 10.1371/journal.pone.0313539 (PMC11819544; doi:10.1371/journal.pone.0313539)
Supplement: S1 Fig — (DOCX) [file pone.0313539.s001.docx]

Figures for correlation of asthma

Figures for correlation of COPD
