# Supplementary material for: Clinical status and cytokine profiles in patients with asthma or chronic obstructive pulmonary disease vaccinated against influenza
Source: PLoS One. 2025 Feb 12;20(2):e0313539. doi: 10.1371/journal.pone.0313539 (PMC11819544; doi:10.1371/journal.pone.0313539)
Supplement: Attachment — Submitted filename: Response Round 2.docx [file pone.0313539.s005.docx]

**1. There remain some methodological concerns that warrant attention. Analyzing binary response and explanatory variables is more effectively conducted through logistic regression, which establishes a curvilinear model relationship, as opposed to a linear probability model. This approach will enhance the manuscript's utility.**

Categorization of spirometry parameters relative to reference values is not informative, since all parameters in 100% of patients are below LLN (lower limit of normal). It is worth noting that Table 2 shows the values as a percentage of LLN, which allows us to evaluate the dynamics of parameters relative to reference values.

A similar situation is also observed with IL2, IL-17 and IL-10 parameters. In all 100% of patients, these parameters did not exceed the upper limit of the reference range throughout the study period. In accordance with the editor's recommendations, this was added to the text of the article.

CRP and IL-6 parameters were categorized relative to the reference range and the dynamics of the proportion of patients with parameter values above the reference were assessed (Table 3, Table 5).

**2.  I am generally satisfied with the responses and updates, and I appreciate the amendments made. However, I would like to remind you to mention the use of multiple corrections testing, update and report the adjusted p-values for both Table 1 and Table 2, and highlight the significant variables based on the adjusted p-values rather than the original p-values. Otherwise, the purpose of performing the Benjamini–Hochberg procedure would be undermined, and your manuscript might contradict your responses to the reviewers (for example, VC, % is still highlighted as significant). This is the only point I suggest modifying further in this revised version.**

Thanks a lot for the remark! Done!
